# Insights Into the Hypometabolic Stage Caused by Prolonged Starvation in L4-Adult *Caenorhabditis elegans* Hermaphrodites

**DOI:** 10.3389/fcell.2020.00124

**Published:** 2020-02-27

**Authors:** E. Carranza-García, Rosa E. Navarro

**Affiliations:** Departamento de Biología Celular y Desarrollo, Instituto de Fisiología Celular, Universidad Nacional Autónoma de México, Mexico City, Mexico

**Keywords:** adult reproductive diapause, oogenic germline starvation response, starvation, stress, germline, germ cells, *Caenorhabditis elegans*, apoptosis

## Abstract

Animals alter their reproductive cycles in response to changing nutritional conditions, to ensure that offspring production only occurs under favorable circumstances. These adaptive strategies include reversible hypometabolic states of dormancy such as “arrest” and “diapause.” The free-living nematode *Caenorhabditis elegans* can arrest its life cycle during some larval stages without modifying its anatomy and physiology until conditions improve but it can also modify its morphological and physiological features to cope with harsh conditions and transition into diapause. The well-defined “dauer” diapause was described more than 40 years ago and has been the subject of comprehensive investigations. The existence of another hypometabolic state, termed adult reproductive diapause (ARD), has been debated after it was first described 10 years ago. Here, we review the current knowledge regarding the effect of food deprivation during the pre-reproductive larval and adult stages on overall organismal homeostasis, highlighting the implications on germ cell maintenance and fertility preservation.

## Introduction

Virtually all animals experience variations in food availability and must adjust their metabolism to survive under harsh conditions. The lack of a food source causes changes in metabolic rates and affects organisms at the sub-cellular level. In a hypometabolic state, biological processes can be slowed or arrested, affecting development, reproduction and gene expression, which in turn results in finely regulated systemic adaptations (Padilla and Ladage, 2012; Baugh, 2013). Many metazoans, ranging from worm to mammals, experience hypometabolic states such as developmental arrest, diapause, quiescence, and hibernation. The nematode *Caenorhabditis elegans* can experience three different types of hypometabolic states throughout its life cycle; embryo suspended animation, larval arrest and diapause. Embryo suspended animation is a reversible hypometabolic state caused by oxygen deprivation and it is characterized by a reduction in the ATP/ADP ratio, which in turn causes an arrest of the cell cycle in the blastomeres (Padilla and Ladage, 2012). Developmental arrest is a reversible hypometabolic state characterized by stress resistance without morphological modifications (Baugh, 2013). In a diapause, animals change their morphology while they wait for conditions to improve. To trigger diapause-specific cue signals for arrest and later for recovery, arrest must occur at a precise stage during the life cycle, and *a priori* changes must occur in the animal metabolism to enable coping with harsh conditions (Kostal, 2006; Androwski et al., 2017).

## *Caenorhabditis elegans* Life Cycle

**FIGURE 1:**
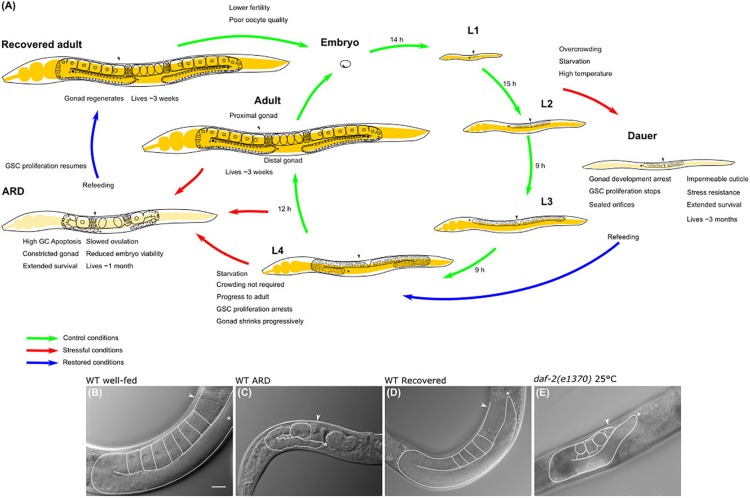
*Caenorhabditis elegans* life cycle and diapauses. **(A)** The life cycle comprises the embryonic stage, 4 larval stages (L1–L4) and the adult stage, which is completed in 3 days. The length of each stage under control conditions is shown in hours (h) (green arrows point toward the transition between larval stages in normal conditions). Life cycle can be reversibly altered at specific checkpoints under harsh conditions to develop into alternate stages termed diapause. Red arrows show the transition from larval stage to diapause, cues triggering the entry into dauer and ARD diapauses and the features of each hypometabolic stage are specified. Blue arrows point to the transition of diapause to recovered conditions. **(B)** Nomarski image of the gonad of a 3-day-old well-fed hermaphrodite. **(C)** Nomarski image of the gonad of an ARD hermaphrodite (starved for 5 days starting from mid L4 stage). Note that these animals produce a single oocyte at a time, which is separated from the rest of the gonad by a constriction. **(D)** Nomarski image of the gonad of a hermaphrodite that was starved for 5 days and recovered in food for 3 days. Its gonad has regenerated and is comparable to the gonad of a young hermaphrodite that never faced starvation. **(E)** Nomarski image of the gonad of a 5-day-old, well-fed *daf-2*(*e1370*) hermaphrodite changed to the restrictive temperature (25°C) at mid L4 larval stage. Well-fed *daf-2*(*e1370*) hermaphrodites grown at 25°C have shrunk gonads that resemble the gonad of an ARD animal; however, their gonads do not form a single oocyte at a time or gonad constrictions. In all images one gonad arm is outlined in white; the distal gonad is marked with an asterisk (*); and the arrow points to the proximal gonad. Scale bar = 20 μm.

*Caenorhabditis elegans* serves as a powerful model for studying the effects of nutrient availability on development and fertility due to its short and well characterized life cycle (Frezal and Felix, 2015). It develops through 4 larval stages (L1–L4) and a reproductive adult stage, and the stages are separated by molts; under laboratory conditions, with plenty of food and at 20°C, the life cycle is completed in approximately 3 days (from hatching to the adult stage) (Figure 1A). Adult hermaphrodites reproduce for 3 days and give rise to ∼260 new organisms through self-fertilization and up to 500 new organisms by mating. After they complete their reproductive period, the remainder of the animals’ life ranges from 12 to 30 days (Pazdernik and Schedl, 2013).

## *Caenorhabditis elegans* Developmental Arrest and Diapause

Lack of food alters the life cycle depending on the stage in which animals face starvation and can result in one of the following; (1) larval arrest or (2) diapause entry (Figure 1A). Animals that hatch under starvation conditions remain viable, arrest their development in the L1 stage, become more resistant to other stresses and can survive for up to 10 days until food is provided (Johnson et al., 1984; Baugh, 2013). Starved L1 larvae show a clear behavioral feature when maintained on solid culture; initially, L1 larvae thrive on the plate in search of food, then larvae aggregate in large groups to obtain small traces of metabolites from the growth medium to ensure their viability in the absence of food, and after some days the aggregates disassociate to form lawns of larvae over the growth medium (Artyukhin et al., 2015). L1 larval arrest and its molecular regulation have been extensively reviewed by Baugh (2013).

L2 animals provided with a limited number of heat-killed bacteria as food remain arrested for up to 9 days (Ruaud and Bessereau, 2006). Additional checkpoints during L3 and L4 exist; when subjected to starvation, larvae arrest the development of specific tissues, thus remaining in a state of dormancy. Animals arrested in the L3 stage survive for up to 9 days, preserving the ability to become adults and live their remaining lifespan when food becomes more abundant (Schindler et al., 2014). When early L4 larvae are deprived of food, they enter an arrest that is characterized by the inhibition of vulval development (Schindler et al., 2014).

As mentioned above, *C. elegans* diapause is characterized by morphological changes that distinguish animals from their well-fed counterparts, and the changes enable them to endure harsh environmental conditions (Hand et al., 2016). Animals subjected to different sorts of stressors at the L1–L2 larval transition, such as limited food availability or a pheromone produced in overcrowding populations, develop into a well-studied alternate L3 larval stage termed “dauer” diapause (Figure 1A; Cassada and Russell, 1975; Golden and Riddle, 1982) [reviewed in detail by Hu (2007) and Calixto (2015)]. During this diapause, animals seal their orifices, produce an impermeable cuticle and halt their reproductive development; these changes allow animals to cope with stress for months, preserving their reproductive capacity once they encounter a food source (Cassada and Russell, 1975).

In this review, we will describe the current understanding regarding the hypometabolic state occurring in the late larval (L4) and adult stages of the nematode *C. elegans*, which is termed the “adult reproductive diapause” (ARD). This hypometabolic state was discovered ten years ago, and there is still so much to learn about its features, how it is induced and regulated. Since the original work was published, many of the original conclusions have changed, and the aim of this review is to summarize and simplify the available information on this subject.

## Animals Can Enter Adult Reproductive Diapause From Early L4 Larval Stage Through Adulthood

The term “adult reproductive diapause” or ARD was coined in 2009 by van Gilst’s group to define an unusual “growth suspension stage” that occurs during late development when animals are exposed to starvation (Figure 1A). They observed that when L4 animals that were already committed to adulthood were exposed to fasting conditions, they arrested their development and delayed their reproductive onset (Angelo and Van Gilst, 2009). Angelo and Van Gilst (2009) reported that animals could spend up to 30 days in ARD without altering their adult life span. It was found that starved animals from L4 or adult are smaller than well-fed adults, and their gonads are shorter relative to the length of the animal (Figures 1A–C). Surprisingly, these features are reversible, and upon refeeding, animals resume their growth and reproductive cycle and live a normal lifespan, serving as a mechanism to ensure reproduction under harsh conditions (Figures 1A,D; Angelo and Van Gilst, 2009; Seidel and Kimble, 2011; Carranza-Garcia and Navarro, 2019).

Angelo and Van Gilst (2009) also reported that ARD entry was optimal if it was induced when the majority of animals were in the mid L4 larval stage, as starving early-L4 animals resulted in L4 arrest and starving late-L4 caused a phenotype called *bagging*. This phenotype occurs when starved animals produce embryos that are retained within the uterus, and when hatching occurs, the offspring causes the death of their progenitor (Angelo and Van Gilst, 2009).

Later, Seidel and Kimble (2011) investigated how L4 hermaphrodites respond to starvation by removing animals from their food source at different intervals from L3/L4 molting (0) to the late L4 larval stage (10 h). Despite their age, they observed that all animals, from early L4 to adulthood, were able to survive the first ten days of starvation if bagging was prevented by interfering with embryonic development (Seidel and Kimble, 2011). They classified animals according to their gonad morphology during the L4 stage and referred to them as “Very” early L4 (0 h), “Early” L4 (2 h), Mid/early” L4 (4 h), “Mid/late” L4 (6 h), “Late” L4 (8 h), and “Very late” L4 (10 h). They observed that animals starved from early L4 succeeded more in developing into adults, and the bagging phenotype occurred less often than in animals starved from late L4. Animals starved beginning at the early L4 molt showed a delay in entry into adulthood, onset of oogenesis and embryo production. In contrast, the vast majority of animals starved from the late L4 stage produced more embryos. Seidel and Kimble (2011) concluded that animals starved from early L4 were more likely to avoid the bagging fate and survive because they produced fewer viable embryos.

## Embryogenesis Continues During Starvation

Angelo and Van Gilst (2009) reported that the embryos produced during ARD enter a state of embryonic arrest. They suggested that these embryos could be arrested for up to 5 days after ARD entry. Seidel and Kimble (2011) addressed the fate of individual animals at day 3 of starvation; those animals contained one or two viable-looking embryos within the uterus, and the embryos exhibited signs of early development. At day 4 of starvation, the embryos had hatched inside the progenitor or seemed inviable, which is normal as judged by the length of embryo development under control conditions (14 h at 20°C). They concluded that embryos produced during prolonged starvation are not arrested and proceed normally through development (Seidel and Kimble, 2011).

## ARD Entering Does Not Require Crowding

In contrast to dauer diapause, ARD does not depend on crowding (Seidel and Kimble, 2011). Seidel and Kimble (2011) subjected two different populations of L4 animals to starvation, one group contained 10,000 and another ∼50 animals. They reported that both populations showed the same features after 10 days of starvation (shrunken oogenic germlines and gonad recovery upon refeeding), suggesting that crowding is not required to induce the starvation response (Seidel and Kimble, 2011).

## The Oogenic Germline Starvation Response

Seidel and Kimble (2011) suggested the term “oogenic germline starvation response” to refer to the germline changes that occur after exposing L4 or adult animals to prolonged starvation. Starved animals exhibit progressive germ cell loss that causes gonad shrinkage (Figures 1A,C). During the first 3 days of starvation, germ cell numbers drop from ∼150 germ cells to a small pool of ∼35 germ cells per gonad arm (Figure 1A; Angelo and Van Gilst, 2009; Seidel and Kimble, 2011, 2015; Carranza-Garcia and Navarro, 2019).

Additionally, the number of germ cells present in the gonad is altered, and the gonad reduces its content dramatically, showing constrictions along the entire gonadal surface, with the only exception being in the distal fraction (Figure 1C; Seidel and Kimble, 2011; Carranza-Garcia and Navarro, 2019). Germ cell loss and gonad shrinkage depend on active ovulations, and wild-type non-oogenic germlines (like those of animals that exhaust their sperm supply) rarely show signs of shrinking (Seidel and Kimble, 2011). Consistently, feminized gonads from mid L4 *fog-1*(*q253*) or *fog-2*(*q71*) mutant animals, which produced several oocytes stacked within their gonads, were unable to reduce their germ cell number during starvation (Cinquin et al., 2016; Carranza-Garcia and Navarro, 2019). Since ovulation practically does not occur in feminized mutant animals, the gonad is not using its resources to produce oocytes and the gonad does not shrink during starvation.

Adult animals typically produce from six to ten oocytes per gonad arm under control conditions (Figure 1B), while starved animals produce only one oocyte per gonad arm. This oocyte is separated from the rest of the gonad by a narrow constriction (Figures 1A,C; Seidel and Kimble, 2011; Carranza-Garcia and Navarro, 2019). Under control conditions, ovulation occurs in each gonad arm every 23 min; by contrast, it occurs approximately every 8 h in starved animals over the first days of starvation, and then by day 10 of starvation, embryo production ceases (Seidel and Kimble, 2011).

Starvation alters the mitotic germ cell cycle as it becomes almost quiescent in the absence of food; phase S of the cycle slows, and most of the germ cells arrest their cycle in the G2 phase but are able to resume their cycle to phase M if conditions are restored. Germ cell cycle arrest depends on the age at which animals are subjected to starvation. The germ cells of young adults slowed down their cell cycle after 30 min of starvation, while those of L4 animals did not halt their cycle immediately; their slowdown occurred once they molted to adulthood (Seidel and Kimble, 2015). Starvation also has an effect on the progression of meiosis; the rate of expression of a meiotic marker (GLD-1) is lower in the gonad of starved animals than in those that were well-fed, suggesting that fasting slows germ cells from progressing into meiosis (Figure 1A; Seidel and Kimble, 2015).

When starved animals were returned to bacteria, their quiescent germ cells resumed their cycle and increased the number of M phase germ cells after 1.5 h (Seidel and Kimble, 2015). Shrunken gonads from starved animals that were refed, regenerated and achieved their original size, and they looked healthy by Nomarski microscopy; for simplicity, we will refer to these animals as *recovered* animals from now on (Figure 1D; Angelo and Van Gilst, 2009; Seidel and Kimble, 2015; Cinquin et al., 2016; Carranza-Garcia and Navarro, 2019).

The mitotic index changed during the time course of the recovery of starved animals. During ARD, the mitotic index was very low (approx. 0.2%), but during the early regeneration period, it increased by approximately 1.5% (Roy et al., 2016). After 72 h, the mitotic index dropped, which correlates with the decline in sperm number. Although germ cell proliferation increases during recovery, it never reaches that of the continuously fed adult animals. The DNA content in the germline was high for the first 24 h, but by 48 h, there was a significant reduction in DNA content that persisted for 96 h (Roy et al., 2016). These results show that although germ cell proliferation after ARD is similar to that of the larval expansion phase, a great proportion of the nuclei that were produced later do not have “wild-type” DNA content, suggesting that some aspects of ARD affect germ cell propagation (Roy et al., 2016). Seidel and Kimble (2011) noticed that regeneration of the gonad is not essential for embryo production. They observed that when unidentified contaminating bacteria grew on the starvation plates, animals fed on them and produced embryos without fully recovering their gonad size.

## Prolonged Starvation Affects Germ Cell Quality

Exposure of mid L4 larvae to 15 days of starvation severely impairs fertility, even when recovered animals are crossed to well-fed males (Angelo and Van Gilst, 2009; Carranza-Garcia and Navarro, 2019). Recovered animals produce fewer progeny than well-fed animals; ∼70% production by self-fertilization is observed in comparison to controls. Although the fertility of animals recovered from 5 days of starvation improved considerably when they were mated with males that never faced starvation, the level of progeny production never reached that of the controls (∼80% of controls). Exposing animals to starvation from mid L4, when spermatogenesis is progressing, does not affect the amount of sperm produced during the L4 stage, as judged by the number of spermatids within the spermatheca. The number of spermatids remained unchanged, at least during the first 5 days of starvation, suggesting that the low progeny numbers produced after ARD are not due to an insufficiency of spermatids; rather, they are due to alterations in the oogenic reproductive capacity (Carranza-Garcia and Navarro, 2019).

Self-fertilizing animals recovered from 5 days of starvation show higher embryonic lethality than animals that never were starved. Unexpectedly, embryonic lethality decreases when recovered animals are crossed with well-fed wild-type males, suggesting that even though sperm exposed to ARD successfully fertilized oocytes, they fail to contribute to successful embryogenesis (Carranza-Garcia and Navarro, 2019). More studies are necessary to understand the effect that starvation could have on spermatids and how this affects sperm contribution to embryogenesis.

Feminized germlines such as those of *fog-1*(*q253*) or *fog-2*(*q71*) mutant animals can be used to avoid the effect of starvation on male gametes. *fog-1*(*q253*) is a temperature sensitive mutation that affects spermatid production when animals are grown at restrictive temperatures; however, this process is unaltered when animals are kept at permissive temperatures (Barton and Kimble, 1990). The *fog-2*(*q71*) mutation affects spermatogenesis only in hermaphrodites, and functional males are needed to cross with feminized *fog-2*(*q71*) mutant animals to maintain this strain (Schedl and Kimble, 1988). The feminized hermaphrodites produce oocytes that arrest in diakinesis for a time that is longer than that of wild-type hermaphrodites because they lack an ovulation signal from sperm. Ovulation in gonads that lack sperm is very low, even in well-fed animals, so oocytes stack in a row within the gonad. An improvement in fertility was observed in *fog-2*(*q71*) feminized hermaphrodites that were exposed to 2 days of starvation (from the L4 stage) and recovered for 24 h before being crossed with males (almost twofold compared to well-fed feminized animals) (Cinquin et al., 2016). In contrast, *fog-1*(*q253*) and *fog-2*(*q71*) feminized hermaphrodites that are exposed to 5 days of starvation and are allowed to recover, for 1 day before being crossed with well-fed males, show a reduction of approximately 50% in their progeny production when compared to that of their controls (Carranza-Garcia and Navarro, 2019). The differences between these two reports could be accounted for as follows: First, the age of animals at the time of initiating starvation; apparently Cinquin et al. (2016) used early L4 animals while Carranza-Garcia and Navarro (2019) used mid L4 animals (Cinquin et al., 2016; Carranza-Garcia and Navarro, 2019). Second, there were differences in the length of the starvation experiments (2 days exposure vs. 5 days, respectively).

Additionally, the embryonic lethality of feminized animals exposed to 5 days of starvation is higher than that of their controls (Carranza-Garcia and Navarro, 2019). Starved feminized animals successfully arrest germ cell proliferation but fail to shrink their gonads due to the very low ovulation rate (Cinquin et al., 2016). It has been observed that oocytes that spend more time arrested in diakinesis and are fertilized do not successfully complete embryogenesis (Andux and Ellis, 2008). Consistently, it was observed that feminized animals that were kept for 5 days under control conditions and were later crossed with males have higher rates of embryonic lethality than those feminized animals that were starved and recovered (Carranza-Garcia and Navarro, 2019). Perhaps during prolonged starvation, the continued production of oocytes, even at a very slow rate, serves as a protective mechanism for oocytes since they do not arrest for long periods in diakinesis.

## Germ Cell Apoptosis Is Very Active During Starvation, and It Protects Germ Cells Quality

Exposing one-day-old adult animals to 6 h of starvation is sufficient to trigger germ cell apoptosis via LIN-35, the ortholog of mammalian retinoblastoma, which inhibits CED-9/Bcl2 expression (Lascarez-Lagunas et al., 2014). While investigating germ cell apoptosis dynamics during prolonged starvation, it was observed that rates of germ cell apoptosis are very high during fasting (five times higher than in well-fed animals). Even though the gonad is completely shrunken by day 5 of starvation, germ cell apoptosis remains highly active over the next 10 days of starvation (Carranza-Garcia and Navarro, 2019). Clearance of dead germ cell occurs normally during prolonged starvation (judged by time-lapse microscopy), suggesting that there is an upregulation of apoptosis, and not a failure of dead cell clearance (Carranza-Garcia and Navarro, 2019).

CEP-1 is the *C. elegans* homolog of p53, a protein essential for induction of apoptosis in the presence of DNA-damage or meiotic failure (Derry et al., 2001; Schumacher et al., 2005; Bailly et al., 2010; Mateo et al., 2016). *cep-1*(*gk138*) animals still showed increased germ cell death during prolonged starvation, suggesting that germ cell apoptosis is not increased by a DNA-damage or meiosis checkpoint (Carranza-Garcia and Navarro, 2019). LIN-35/Rb-defective animals also showed a slight increase in germ cell apoptosis during prolonged starvation; however, the level of germ cell apoptosis did not reach that of the control animals, suggesting that LIN-35/Rb is partially responsible for triggering germ cell apoptosis during ARD. Unexpectedly, there is an additional mechanism that contributes to germ cell apoptosis during ARD (Carranza-Garcia and Navarro, 2019).

Caspase CED-3 is required for germ cell apoptosis under control and starvation conditions (Salinas et al., 2006; Andux and Ellis, 2008). The van Gilst’s group reported that during ARD, the gonads of *ced-3*-deficient animals did not shrink and/or regenerated upon refeeding, and the authors suggested that apoptosis is required for gonad remodeling during prolonged starvation and recovery (Angelo and Van Gilst, 2009). Using different alleles of *ced-3*-deficient animals (*n1286*, *n717* and *ok2734*), Carranza-Garcia and Navarro (2019) showed that caspase-deficient animals had shrunken gonads when subjected to 5 days of starvation and regenerated their germlines upon refeeding, demonstrating that apoptosis is not important for the gonad shrinking or regeneration process (Carranza-Garcia and Navarro, 2019).

Andux and Ellis (2008) reported that *ced-3* mutant animals produce fewer offspring and exhibit higher embryonic lethality than wild-type animals under control conditions (Andux and Ellis, 2008). Exposing *ced-3* mutant animals to 5 days of starvation further affects their fertility. Recovered *ced-3* mutant animals presented a threefold increase in embryonic lethality when compared to wild-type recovered animals, suggesting that apoptosis is important for maintaining oocyte quality during prolonged starvation (Carranza-Garcia and Navarro, 2019).

## Recovering From ARD

Studying the mechanisms of ARD exit, Burnaevskiy et al. (2018) recovered 3-week-starved animals on food containing the DNA synthesis inhibitor 5-fluoro-2′-deoxyuridine (FUDR). Unexpectedly, animals exposed to FUDR were unable to restore their body and gonad size, normal pumping rate or thrashing (lateral swimming when worms were placed in liquid) after ARD. However, FUDR did not impact adult longevity (Burnaevskiy et al., 2018).

Although the germline can influence *C. elegans* longevity (Arantes-Oliveira et al., 2002), ARD animals lacking a germline [such as *glp-1*(*e2141*)] were able to enter and exit ARD, suggesting that germline regeneration is not implicated in somatic recovery. However, ARD animals lacking a germline did not recover their full size when grown in FUDR (Burnaevskiy et al., 2018).

Burnaevskiy et al. (2018) found that progression from L4 to adulthood during prolonged starvation occurs normally, and somatic cell divisions or endoduplications are not postponed. Using *cye-1*(*eh10*) mutant animals (cyclin E-deficient) that were subjected to prolonged starvation and then were refed, Burnaevskiy et al. (2018) found that cell cycle reactivation in somatic tissues is not required for post-ARD recovery. To test whether the inhibition of mitochondrial DNA replication might underlie the inhibitory effects of FUDR on post-ARD recovery, animals that lack the mitochondrial DNA polymerase *polg-1*(*ok1548*) were exposed to starvation and then were allowed to recover from ARD. These animals showed normal recovery and unaltered mitochondrial DNA content, leading to the conclusion that mitochondrial DNA synthesis is not required for soma recovery from ARD. To test whether FUDR affects post-ARD recovery by inhibiting RNA metabolism, media was supplemented with extra DNA or RNA nucleotides. Adding uracil, uridine or deoxyuridine, but not thymidine, rescued the effects of FUDR on somatic restoration (Burnaevskiy et al., 2018). Similar results were obtained when animals were fed with bacterial mutant strains deficient in nucleotide-processing enzymes such as thymidine-kinase (strain JW1226-1, Δ*tdk*) or uracil phosphoribosyltransferase (strain JW2483-1, Δ*upp*). In addition, worms carrying a temperature-sensitive allele of thymidine synthase [*tyms-1*(*hc65*)] recover normally from ADR at the restrictive temperature. TYMS-1 is one of the targets of FUDR that mediates its DNA-based toxicity (Burnaevskiy et al., 2018).

Additionally, Burnaevskiy et al. (2018) found that ARD animals exhibit smaller nucleoli than their well-fed counterparts. Upon refeeding the animals, their nucleoli increased in size, reaching a size that was comparable to that of young adults under control conditions. However, FUDR affects the structure of nucleoli in control animals, and this defect was partially rescued when the food source was supplied with uracil. FUDR impairs expansion of total RNA upon exit from ARD, and this effect was mostly suppressed by supplementation with uracil (Burnaevskiy et al., 2018).

RNA content importantly increases upon recovery and FUDR apparently prevents post-ARD recovery through inhibition of RNA metabolism. However, FUDR treatment affects the RNA amounts, not only in recovered animals but also in well-fed control animals; therefore, it is important to note that this drug interferes with global RNA metabolism (Burnaevskiy et al., 2018).

## The Molecular Mechanisms Governing the Starvation Response

Angelo and Van Gilst (2009) reported that the transcription factor *nhr-49*/HN4F regulates gonad reduction during prolonged starvation and fertility recovery upon refeeding (Table 1; Angelo and Van Gilst, 2009). They also reported that *ced-3* mutant animals did not show shrink gonads and did not recover after ARD, suggesting that apoptosis is important for ARD (Angelo and Van Gilst, 2009). Later, it was reported that gonad shrinkage during starvation and gonad recovery upon refeeding do not depend on apoptosis; therefore, the oogenic starvation response is not regulated by apoptosis (Table 1; Carranza-Garcia and Navarro, 2019). Consistently, LIN-35/Rb, which partially regulates starvation-induced germ cell apoptosis, is not necessary for gonad shrinking or recovery after ARD (Table 1; Carranza-Garcia and Navarro, 2019).

**TABLE 1:** Genes that have been tested for ARD regulation.

Gene	Soma	Germline	Developmental stage	Starvation length	References
*ced-3*/Caspase	ND	NR	Mid L4	5 and 15 days	Carranza-Garcia and Navarro, 2019
*glp-1*/Notch receptor	NR	ND	Early mid L4	3 weeks	Burnaevskiy et al., 2018
*polg-1*/Mitochondrial DNA polymerase	NR	R	Early mid L4	3 weeks	Burnaevskiy et al., 2018
*tyms-1*/Thymidine synthase	NR	NR	Early mid L4	3 weeks	Burnaevskiy et al., 2018
*cye-1*/Cyclin-E	NR	ND	Early mid L4	3 weeks	Burnaevskiy et al., 2018
*daf-2*/InsR	NR	NR*	Mid L4	5 days	Carranza-Garcia and Navarro, 2019
*daf-16*/FoxO	NR	NR	Mid L4	5 days	Carranza-Garcia and Navarro, 2019
*prg-1*/*Piwi*	ND	ND*	–	–	Heestand et al., 2018
*nhr-49*/HNF4α	R	R	Early mid L4	15 days	Angelo and Van Gilst, 2009
*lin-35*/Rb	ND	NR	Mid L4	5 days	Carranza-Garcia and Navarro, 2019
*pha-4*/FoxA	ND	NR	Mid L4	5 days	Carranza-Garcia and Navarro, 2019
*skn-1*/Nrf	ND	NR	Mid L4	5 days	Carranza-Garcia and Navarro, 2019
*cep-1/p53*	ND	NR	Mid L4	5 days	Carranza-Garcia and Navarro, 2019
*alg-1/Argonaut*	ND	NR	Mid L4	5 days	Carranza-Garcia and Navarro, 2019
*rsks-1/S6K* protein	ND	NR	Mid L4	5 days	Carranza-Garcia and Navarro, 2019
*ife-1/eIF4e*	ND	NR	Mid L4	5 days	Carranza-Garcia and Navarro, 2019
*gla-3/TTP*	ND	NR	Mid L4	5 days	Carranza-Garcia and Navarro, 2019

R, required; NR, not required; ND, not determined; d, days. *Mimics some aspects of ARD.

Searching for factors that might regulate the oogenic starvation response during the adult diapause, mid L4 animals from different genetic backgrounds were subjected to prolonged starvation. None of the key regulators of dauer diapause and the L1 arrest, such as *pha-4*/FoxA, *daf-16*/FoxO, *skn-1*/Nrf, were required for gonad shrinking during starvation or recovery upon refeeding (Table 1; Carranza-Garcia and Navarro, 2019). Importantly, whether these regulators are required to orchestrate other aspects regarding the soma of ARD animals remains to be tested.

The IIS pathway in *C. elegans* is controlled by the insulin receptor *daf-2* and transcriptional factor *daf-16*/FoxO (Lee et al., 2003). When mid L4 *daf-16*(*mgDf50*) mutant animals were subjected to prolonged starvation, they exhibited progressively reduced gonads that recovered in size after ARD, which was equivalent to their wild-type counterparts and indicated that this pathway is dispensable for the oogenic germline starvation response (Table 1; Carranza-Garcia and Navarro, 2019). Unexpectedly, well-fed *daf-2*(*e1370*) animals at restrictive temperature have a shrunken gonad that shows some but not all of the characteristics of ARD germlines, such as the following: (1) they progressively reduce their germline from L4 to adulthood and (2) they have a decreased rate of ovulation. However, they did not produce a single oocyte at a time during prolonged starvation or produce gonad narrowing behind the oocytes (Figure 1E; Carranza-Garcia and Navarro, 2019). Other aspects of ARD in the soma remain to be tested in *daf-2*(*e1370*) animals.

Heestand et al. (2018) proposed that *prg-1/Piwi* mutants become sterile via a mechanism that resembles ARD (Table 1; Heestand et al., 2018). PRG-1 is the Piwi homolog in *C. elegans*. Piwi and its Piwi-interacting RNAs (piRNAs) promote transgenerational genomic silencing and germ cell mortality. *prg-1/Piwi* mutants have a transgenerational fertility defect that can be suppressed by reduction of the insulin/IGF1 stress response pathway. In the eighteenth generation, *prg-1* mutant animals have small gonads and high levels of apoptosis when they are fed OP50 bacteria. The gonad size of *prg-1/Piwi* mutants, regenerates after feeding animals with a different bacterial strain (HT115) (Heestand et al., 2018).

Apoptosis and necrosis contribute to germline atrophy in *prg-1* mutant animals. However, apoptosis is not required for gonad shrinking during ARD (Carranza-Garcia and Navarro, 2019); therefore, in this aspect *prg-1* mutant animals do not resemble the oogeneic germline starvation response. Furthermore, DAF-16 rescues germline atrophy in *prg-1* animals (Heestand et al., 2018) but this molecule did not contribute to germline shrinking during ARD (Carranza-Garcia and Navarro, 2019). Additionally, gonads from *prg-1* mutant animals do not produce a single oocyte at the time or produce gonad narrowing behind the oocytes, which are present in ARD animals. Although it is possible that *prg-1* mutant animals enter a form of ARD, more experiments need to be done to demonstrate this. Other genes that have been tested but are not required for ARD or the oogenic germline starvation response are listed in Table 1.

## Conclusion and Raised Questions

Adult reproductive diapause was reported 10 years ago, and some of the initial observations have either been studied in depth and confirmed or have been corrected. Because there exist some discrepancies in the results obtained from similar experiments, it is of great significance to establish general considerations when studying this hypometabolic state. Published works have focused on both soma and germline responses to prolonged starvation of L4 and adult animals, while other works have focused mainly on the soma response; still others have focused exclusively on the germline response (oogenic germline response). It is not yet clear if there is a common regulator of the soma and germline response during ARD; therefore, it is important to define what attributes of the ARD will be studied.

It is also important to establish the age at which animals will be exposed to starvation because different results can be obtained if animals are exposed to starvation at L4, mid L4 or adulthood. Another aspect that is worth considering is the time that animals will spend in starvation conditions. If studying the oogenic germline response, it is important to know that gonad shrinking is completed at 5 days of fasting; however if starvation is prolonged for 15 days, animals will not recover their fertility after ARD.

To avoid confusion, we used the term ARD to refer to the hypometabolic stage that occurs when L4 or adult animals are exposed to prolonged starvation. Whether ARD should be considered a diapause or not remains to be elucidated. Some distinguishing features of diapause indicate ARD is different. For example, the main purpose of a diapause is to preserve species continuity under harsh conditions, but prolonged starvation results in infertility after 15 days of starvation. All L4 stage animals respond equivalently by progressing their lifecycle into adulthood, but they do so at different rates. L4 or adult animals do not prepare themselves by modifying their anatomy as the dauer diapause. However, we cannot rule out the possibility that it could be a form of diapause, since starvation is the main cue triggering this hypometabolic stage, and this mechanism seems to protect animals from aging because they continue the rest of their lifespan after re-feeding.

Although studies regarding the starvation response have focused mainly on oogenic germ cells, the effect of starvation on males and hermaphrodite male germ cells remains unclear. Additionally, more investigation needs to be done on the transcriptional profile of animals during ARD to compare it to that of the dauer larva and delineate the precise regulation guiding ARD entry and exit. If ARD has effects on fertility and oocyte quality, it would be of great interest to determine the transgenerational effects of the offspring produced during and after ARD, not only at the epigenetic level but also to the organismal extent, by measuring features such as body size, lifespan, progeny production and stress resistance, to mention some aspects. Complementary studies on the quality of gametes produced after ARD; such as the mRNA content within the oocytes, oocyte size, and embryonic development timing, among many others, would reveal the mechanisms altered by starvation, which promote reproduction under harsh conditions and may be used as biomarkers of aging or reproductive capacity. Another point of interest is the identification of molecules involved in starvation-induced germ cell apoptosis regulation during ARD. This knowledge should enhance our understanding of the preservation of fertility and oocyte quality. It will be interesting to see how ARD promotes survival under starvation conditions and whether this metabolic state of dormancy confers some type of stress resistance.

## Author Contributions

EC-G and RN reviewed and wrote the manuscript. EC-G made the figure.

## Conflict of Interest

The authors declare that the research was conducted in the absence of any commercial or financial relationships that could be construed as a potential conflict of interest.
